# Anthropogenic threats to owls: Insights from rehabilitation admittance data and rodenticide screening in Texas

**DOI:** 10.1371/journal.pone.0289228

**Published:** 2023-08-04

**Authors:** Eres A. Gomez, Heather L. Prestridge, Jennifer A. Smith

**Affiliations:** 1 Department of Integrative Biology, The University of Texas at San Antonio, San Antonio, Texas, United States of America; 2 Biodiversity Research and Teaching Collections, Department of Ecology and Conservation Biology, Texas A&M University, College Station, Texas, United States of America; University of Regina, CANADA

## Abstract

Owls (Strigiformes) provide myriad ecosystem services and are sentinels for ecosystem health. However, they are at continued risk from anthropogenic threats such as vehicle collisions, entanglement with human-made materials, and exposure to anticoagulant rodenticides (ARs), a widespread pesticide known to affect owls. Texas is an important region for numerous migratory and non-migratory owl species in the United States (US), yet assessments of threats owls face here are lacking preventing the development of informed conservation strategies. This study coupled assessment of admittance data from two wildlife rehabilitation centers in Texas with AR liver screening to (1) identify which species of owls are commonly admitted, (2) evaluate seasonality of admittance, and (3) assess causes of admittance for owls in Texas. Between 2010 and 2021, 1,620 owls were admitted into rehabilitation, representing eight species of which the Great-horned Owl (*Bubo virginianus*) was the most common. For all owls combined admittance rates were highest in the spring, driven by an influx of juveniles (n = 703, 43.40%). The leading cause of admittance amongst species was ‘no apparent injury’ (n = 567, 34.94%). Where clear diagnoses could be made, the leading causes of admittances were ‘entrapment in human infrastructure’ (n = 100, 6.11%) and ‘collision with vehicles’ (n = 74, 4.56%). While the admittance data did not reveal any cases of AR poisoning, liver screening demonstrated high incidences of AR exposure; of 53 owls screened for ARs, 50.94% (n = 27) tested positive with 18 showing exposure to multiple ARs. Brodifacoum was the most frequently detected AR (n = 19, 43.18%) and seven owls (25.93%) tested positive within lethal ranges. Our results suggest that owls in Texas are at risk from myriad anthropogenic threats and face high exposure rates to ARs. In doing so, our results can inform conservation strategies that mitigate anthropogenic threats faced by owls in Texas and beyond.

## Introduction

Owls (Strigiformes) are a widespread raptorial group of birds in North America [1], many of which regulate rodent populations, and thus may be important for minimizing damage to crops and human structures and for providing disease control [2–4]. Furthermore, many species of owls are top predators, and thus are valuable sentinels of ecosystem health, highlighting the importance of their conservation [5]. However, in North America owls face numerous anthropogenic threats including collisions with vehicles, entanglement with man-made materials (e.g., fishing wire or kite string), electrocution, and poisoning [6–8]. Such threats are likely to increase as human populations in North America continue to grow, with the potential for owl populations to decline in the absence of conservation efforts [9–11]. Thus, there is an urgent need to increase knowledge of the anthropogenic threats faced by owls in this region so that informed conservation strategies that mitigate losses due to anthropogenic activities can be developed.

Recent attention has highlighted the potential for anticoagulant rodenticide (ARs) poisoning, a widespread issue increasingly recognized as a global conservation concern for owls to have effects at the population level [12]. ARs are used to control pest rodent populations and include First Generation Anticoagulant Rodenticides (FGARs) and Second Generation Anticoagulant Rodenticides (SGARs), the latter of which are more toxic to wildlife [13]. Exposure to ARs occurs primarily via secondary exposure when an owl consumes poisoned prey and may cause myriad effects including organ failure, internal and/or profuse bleeding from open wounds, and increased risk to injury, disease, and predation [12]. Yet, our knowledge of AR exposure rates in owls is limited in part due to the difficulty in confirming AR poisoning via postmortem examination, because of challenges associated with diagnostic testing, and because of geographic biases present in existing studies [12, 14]. For example, in North America, assessments of AR exposure in owls have largely been confined to Canada [15–18], California [19, 20], and the Atlantic Northeast [21–23]. Because incidences of AR exposure appear to be both site- and species-specific [12], further research focused on AR exposure in concert with other anthropogenic threats is needed from understudied regions to inform conservation efforts.

Rehabilitation admittance data collected following admittance of owls into wildlife rehabilitation centers provides valuable insight into the anthropogenic threats they face. For example, previous studies using rehabilitation data suggest owls are frequently admitted following barbed-wire fence injuries and collisions with vehicles or other manmade structures [24–26]. Concomitantly, rehabilitation admittance data provides insight into the species most likely to be admitted and when they are most vulnerable to anthropogenic threats [7, 25, 26]. However, a lack of access to resources (e.g., lab equipment and money to process samples) often prevents wildlife rehabilitation centers from confirming AR exposure via diagnostic tests. As a result, rehabilitation admittance data may underestimate AR exposure rates. Nevertheless, such data has proven valuable in identifying key threats to owls worldwide [24, 27–29]. In North America, studies using rehabilitation admittance data have largely been restricted to Canada or the northern United States (US) [7, 30, 31]. Further, to our knowledge, such studies have not integrated diagnostic testing to quantify AR exposure alongside other threats.

Texas is the second largest state (~695,622 km^2^) within the US with a human population of 29 million [32]. It is ecologically diverse, encompassing ten ecoregions ranging from the arid and mountainous region of the Trans-Pecos in the west, to the Piney Woods in the east [33]. Due to the diversity of ecoregions, the state is important for numerous migratory and non-migratory owl species of which 11 occur annually on a regular basis [34]. At the same time, Texas also hosts some of the largest and fastest growing cities in the nation where owls may be increasingly at risk from building collisions, persecution, and AR poisoning [24, 35]. However, despite the importance of Texas for owls, assessments of threats they face in this region are lacking hindering the development of informed conservation plans. The objectives of this study were to use admittance data from wildlife rehabilitation centers to (1) identify which species of owls are commonly admitted, (2) evaluate seasonality of admittance, and (3) assess causes of admittance for owls in Texas. We predicted (1) that Barred Owls (*Strix varia*) and Great-Horned Owls (*Bubo virginianus*) would be admitted most often because they are relatively abundant compared to other species in Texas (S1 Table), (2) that admittances would be higher in the summer and spring due to an increase in young owls being mistaken as orphans during this period [26], and (3) the leading cause of admittance would be injury due to vehicle collisions and bodily injury from unknown causes based on previous studies [24–26]. In addition, we used liver analysis to estimate the prevalence of AR exposure of owls in Texas. We predicted that liver analysis would reveal high incidences of AR exposure because ARs are widely used [35] and small mammals are a main prey item of the majority of owl species in Texas [34].

## Materials and methods

### Rehabilitation admittance data

Raptor rehabilitation admission records for owls rescued within Texas (centered on: 29°44’52.728”, -98°30’18.8352”) were collected from two wildlife rehabilitation centers: Last Chance Forever the Bird of Prey Conservancy (LCF) and Wildlife Rescue & Rehabilitation, Inc. (WRR). While these facilities primarily receive owls from south and central Texas, birds from across the state are admitted. Further, the risks faced by owls in the primary areas served by the facilities (i.e. south and central Texas) are likely consistent with those faced by owls at the state level because south and central Texas encompass myriad land covers and uses, and thus present a broad range of threats to owls. Thus, the data likely reflect challenges experienced by owls at the state level. Data from LCF were provided electronically, representing owls admitted between January 2010 and December 2020 whereas data from WRR were extracted from the Wildlife Rehabilitation Medical Database (WRMD; wrmd.org), representing owls admitted between January 2016 and December 2021. Data collected included species, date admitted (i.e., year and month), and cause of admittance based on visual observations of the body or details supplied by the caller or rescuer. All owls were identified at the species level, except Eastern Screech Owl (*Megascops asio*) and Western Screech Owl (*Megascops kennicottii*), which were grouped together as Screech Owl (*Megascops* spp.). Owls were classified as either juvenile or adult based on plumage characteristics [36].

For the purpose of this study, each owl was assigned to one of 14 cause of admittance categories (Table 1) based on primary diagnosis upon intake (i.e., visible clinical signs and nature of injury) as follows: (1) bodily injury (cause unknown), (2) eye injury (cause unknown), (3) collision with vehicle, (4) collision with human infrastructure (e.g., buildings), (5) entrapment with human infrastructure (e.g., barbed wire fence or fishing line), (6) electrocution, (7) contaminant exposure (e.g., lead, motor oil, or unknown), (8) anticoagulant rodenticide (AR) poisoning, (9) neurological distress (cause unknown), (10) persecution (e.g., shot with airguns, firearms, or bow), (11) attacked or caught by domestic pet (i.e., dog or cat), (12) disease (e.g., avian pox, trichomoniasis, aspergillosis, etc.), (13) no apparent injury, and (14) undiagnosed. Apparently healthy orphaned nestlings and fledglings admitted due to human intervention or nest removal were categorized as ‘no apparent injury’. Individuals transferred from another rehabilitator for further examination without accompanying information or that exhibited unexplained lethargy/dehydration/emaciation with no other signs of injury were categorized as ‘undiagnosed’.

**Table 1:** Definitions of the 14 categories used to assign cause of admittance for owls admitted to rehabilitation between 2010 and 2021. Owls were admitted to two facilities that serve Texas, US (Last Chance Forever the Bird of Prey Conservancy and Wildlife Rescue & Rehabilitation, Inc.).

Admission Cause Category	Definition
Bodily Injury (cause unknown)	Broken bones and miscellaneous injuries (cause unknown); includes open wounds and superficial scrapes, cuts, abrasions, and/or bruising.
Eye Injury (cause unknown)	Injury to eyes (e.g., blood present, torn ocular tissue, foreign object in eyes, lack of pupillary reflex, or blindness).
Collision with Vehicle	Collision with moving object (e.g., car or truck); sometimes witnessed, other times animal retrieved from vehicle parts (i.e., grill), or suspected collision with vehicle based on presenting symptoms/injuries (e.g., broken legs, wings, fractured pelvis or back) and location of rescue (i.e., found on side of road).
Collision with Human Infrastructure	Collision with stationary human infrastructure (e.g., buildings, glass windows, towers, or powerlines); sometimes witnessed visually or audibly; includes suspected collision with stationary human infrastructure based on presenting symptoms/injuries (e.g., broken wings or coracoid, cracked beak, or stunned behavior) and location of rescue (i.e., found on the ground near side of building).
Entrapment with Human Infrastructure	Entanglement with anthropogenic material (e.g., fishing equipment, barbed wire fence, string, or netting) and entrapment inside human-made structures (e.g., buildings, chicken coop, or swimming pool).
Electrocution	Suspected electrocution based on characteristic presenting symptoms (e.g., electrical burns, degloving, burnt odor, visual observation of electrical current entry and exit wounds, charred skin or feathers).
Contaminant Exposure	Confirmed exposure to lead (through diagnostic testing or visualization of fragments) or motor oil.
Anticoagulant Rodenticide (AR) Poisoning	Suspected exposure to blood-thinning compounds (e.g., warfarin, brodifacoum, bromadiolone, difenacoum, and diphacinone). Presenting symptoms include anemia and severe bleeding and/or hemorrhage.
Neurological Distress (cause unknown)	Birds exhibit signs of nervous system disorders and may present with difficulty standing or leaning to one side, head tilt, abnormal pupillary light response or menace (blink reflex), dazed state or impaired consciousness, ataxia (inability to control muscle movement), seizures or paralysis.
Persecution	Intentional harm or capture (e.g., shooting or trapping) by a human typically diagnosed through examination of injuries (e.g., shattered bones or entry wounds), witness testimony, retrieval of ammunition fragments during surgery or necropsy, or by visualizing fragments with radiographs.
Attacked or Caught by Domestic Pet	Injuries sustained by interspecific interaction with domestic pets (e.g., dog or cat), sometimes witnessed by pet owner or a concerned community member and diagnosed by observation of resulting injuries (e.g., bite marks or claw punctures). Some cases include displaced, but unharmed eggs, nestlings, or fledglings by domestic dog.
Disease	Illness of a pathologic origin including bacterial, viral, or fungal infectious disease or parasitosis (e.g., Avipoxvirus [avian pox], Trichomoniasis [frounce], Aspergillosis [asper], Coccidiosis, Botulism, West Nile Virus, torticollis, bumblefoot, sour crop).
No Apparent Injury	Healthy individuals with no obvious injuries. Includes displaced juveniles (e.g., nestlings, fledglings, or branchers) admitted during nesting season that have either fallen out of nests or were unnecessarily abducted, and removal or relocation due to public nuisance.
Undiagnosed	Individuals admitted due to unknown causes, showing signs of unexplained lethargy, dehydration, malnutrition, or emaciation; birds transferred from another rehabber for further examination and rehabilitation without information.

### Data analysis

We determined frequencies of admittance by species, season (spring [March-May], summer [June-August], fall [September-November], and winter [December-February]), and for each cause of admittance for all species combined and for each species of owl represented in the dataset. Chi-square goodness-of-fit tests were performed to determine if observed counts based on species, admittance type, and season differed from expected. Expected counts were considered equivalent amongst categories for the admittance type and season analyses. For the species analysis, we used population estimates obtained from the Partners in Flight Database [37] to calculate expected counts amongst species (Short-eared Owls [*Asio flammeus*] and Long-eared Owls [*Asio otus*] were omitted from this analysis due to population estimates being unavailable). Standardized residuals were calculated to determine statistical significance (significance was considered where standardized residuals > 2). The analysis described above was performed using two datasets, one that considered all individuals (hereafter ‘full dataset’) and one that omitted apparently healthy orphaned nestlings and fledglings categorized as ‘no apparent injury’ (hereafter ‘reduced dataset’). Statistical procedures were performed using the base package of R version 4.1.2.

### Liver sampling

Whole fresh liver samples of at least 2.5 g were opportunistically collected from deceased owls admitted into rehabilitation at LCF and WRR. Additional livers were obtained from owl specimens housed in The Biodiversity Research and Teaching Collections at Texas A&M University (TAMU), College Station, Texas, US or from carcasses opportunistically salvaged from the wild in Texas. All livers were obtained from owls collected in Texas between July 2020 and November 2022. All birds admitted to rehabilitation centers were either dead on arrival, died within the first two weeks of admittance, or were euthanized due to the severity of injuries by rehabilitation staff immediately following admittance. Liver samples were excised and stored in either a sterile 50-mL plastic polypropylene screw-top tube or wrapped in aluminum foil, and frozen at -15°C until transport for processing. All specimens were handled under approved state and federal research permits (USFWS MB89179D; USFWS MB89966C; TPWD SPR-1020-153; TPWD SPR 0720–091) and IBC approved protocol (IBC#B192). No Institutional Animal Care and Use Committee (IACUC) approvals were needed because liver samples were collected after owls had expired.

### Anticoagulant rodenticide analysis

Owl livers were tested in four separate batches between June 2021 and June 2023. The first three batches of livers were tested for four First Generation Anticoagulant Rodenticides (FGARs) (i.e., warfarin, diphacinone, chlorophacinone, and coumatetralyl), and four Second Generation Anticoagulant Rodenticides (SGARs) (i.e., brodifacoum, bromadiolone, difenacoum, and difethialone). The last batch of livers were also screened for an additional FGAR, coumachlor which was added to the test protocol by the testing facility in 2023. All samples were analyzed using liquid chromatography mass spectrometry and the QuEChERS method [38] at the Texas A&M Veterinary Medical Diagnostic Laboratory (TVMDL) in College Station, Texas, US. The limit of detection (LOD) was set at 0.10 ng/g and the limit of quantification (LOQ) was 1.00 ng/g for each compound. AR residues present in trace amounts too small to quantify were assigned a result of <1.00 ng/g [39, 40].

## Results

### Rehabilitation data

**Fig 1:**
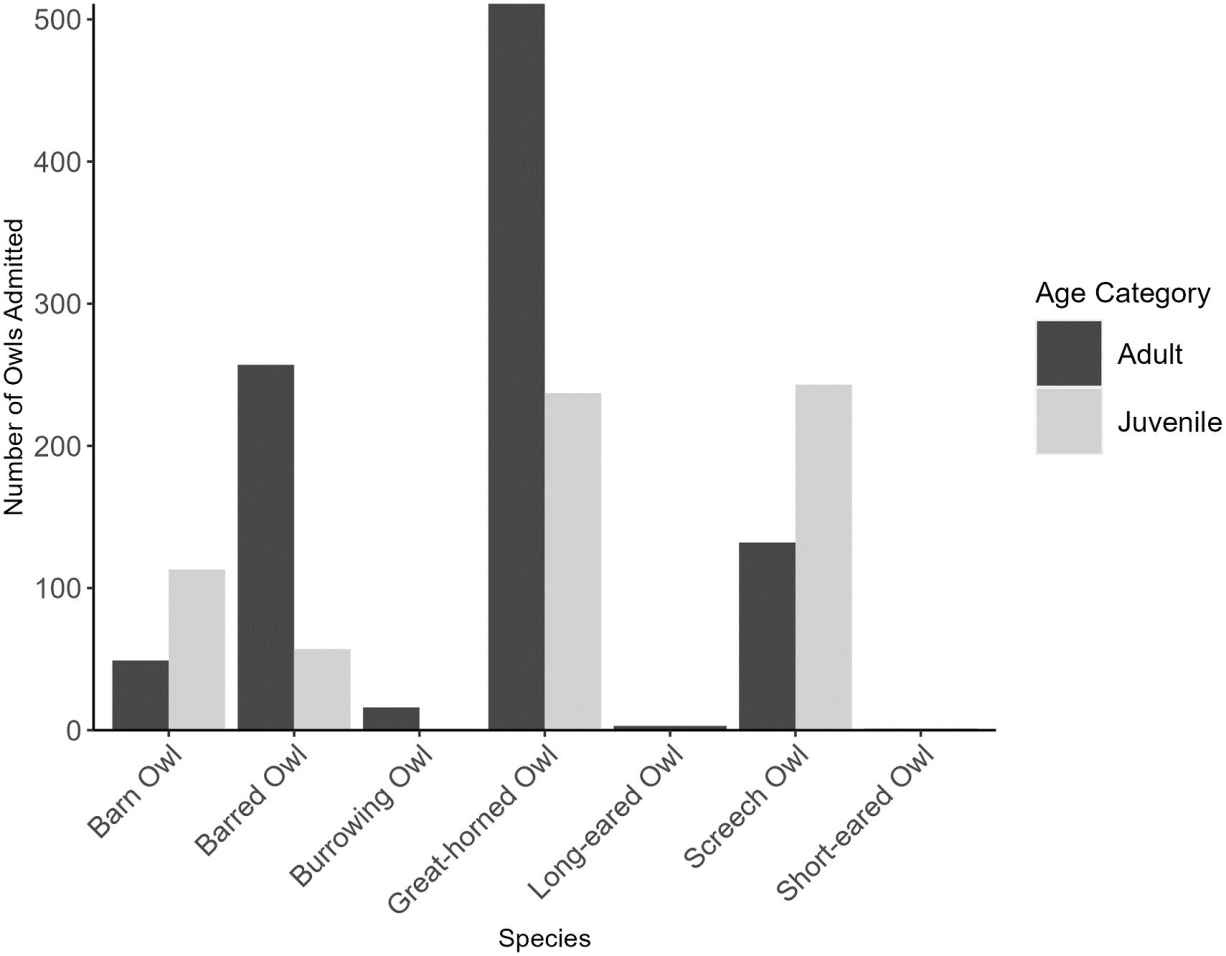
Number of owls admitted per species into rehabilitation between 2010 and 2021. Owls were admitted to two facilities that serve Texas, US (Last Chance Forever the Bird of Prey Conservancy and Wildlife Rescue & Rehabilitation, Inc.). Dark grey bars represent adults and light grey bars represent juveniles.

A total of 1,620 admissions records were collected from LCF and WRR between January 2010 and December 2021 with an average of 135 individuals admitted per year (range: 60–224, SD: 59.24). Admitted owls represented seven species: Great-horned Owl (n = 748, 46.17%), Screech Owl (n = 375, 23.15%), Barred Owl (n = 314, 19.38%), Barn Owl (*Tyto alba*) (n = 162, 10%), Burrowing Owl (*Athene cunicularia*) (n = 17, 1.05%), Long-eared Owl (n = 3, 0.19%), and Short-eared Owl (n = 1, 0.06%) (Fig 1). The observed admittance counts per species were significantly different from expected based on population estimates (*X*^2^ = 1,113.40, df = 4, P<0.0001) with Barn Owls and Screech Owls being admitted more than expected (standardized residuals = 10.43, and 27.09, respectively). In contrast, Barred and Burrowing Owls were admitted significantly less than expected (standardized residuals = -8.02 and -14.30, respectively). Interestingly, Great-horned Owls were admitted close to the expected count (standardized residual = 1.34) based on population estimates for this species [37]. Of the owls admitted, 651 (40.19%) were juveniles and 969 (59.81%) were adults. However, the ratio of adults to juveniles varied amongst species (Fig 1) with Barn Owls and Screech Owls having proportionally higher juvenile admittance rates than other species (n = 113 [69.75%] and n = 243 [64.80%], respectively). In comparison, no juveniles were admitted for both Long-eared and Short-eared Owls.

**Fig 2:**
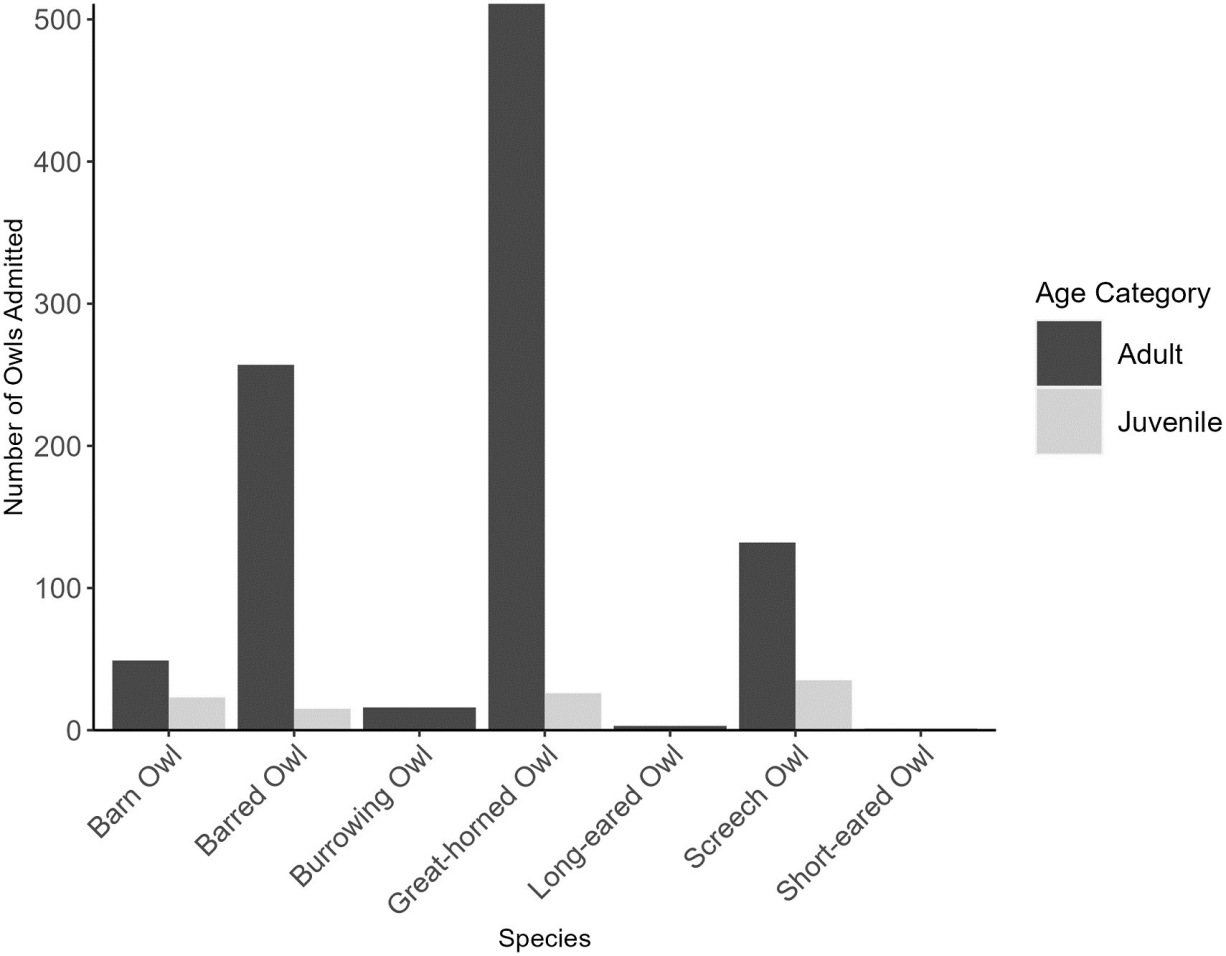
Number (mean ± 1SD) of owls admitted by season into rehabilitation between 2010 and 2021. Owls were admitted to two facilities that serve Texas, US (Last Chance Forever the Bird of Prey Conservancy and Wildlife Rescue & Rehabilitation, Inc.) by season. Dark grey bars represent adults and light grey bars represent juveniles.

The majority of owls were admitted during spring (n = 703, 43.40%), followed by summer (n = 347, 21.42%), fall (n = 310, 19.14%), and winter (n = 260, 16.05%) (Fig 2). Seasonal admittance counts were significantly different from expected (*X*^2^ = 301.77, df = 3, P<0.0001) with more owls being admitted in spring than expected (standardized residual = 14.81), and fewer owls being admitted in fall, summer, and winter than expected (standardized residuals = -4.72, -2.88, and -7.21, respectively). At the species level, this pattern was consistent for Barred, Great-horned, and Screech Owls. However, significantly more Barn Owls were admitted in fall than expected, while significantly fewer were admitted in the winter (*X*^2^ = 17.61, df = 3, P<0.001, standardized residuals = 2.59 and -2.44, respectively). We observed similar results for Burrowing Owls, but caution interpretation due to small sample sizes (n = 8, *X*^2^ = 20.88, df = 3, P<0.001, standardized residuals = 3.76 [fall]).

For all owl species combined, the most common cause of admittance was ‘no apparent injury’ (n = 564, 34.81%), followed by ‘bodily injury (cause unknown)’ (n = 478, 29.51%) and ‘undiagnosed’ (n = 235, 14.51%). In contrast, few admissions were due to ‘contaminant exposure’ (n = 7, 0.43%), ‘neurological distress’ (n = 5, 0.31%), or ‘persecution’ (n = 1, 0.06%). From the admissions data, no owls were admitted due to AR poisoning. These general trends were consistent amongst species (Table 2). Counts of admissions for the different causes for all species combined were significantly different from expected (*X*^2^ = 3,763,70, df = 13, P<0.0001). The greatest differences between observed and expected counts occurred for ‘no apparent injury’, ‘bodily injury (cause unknown)’, and ‘undiagnosed’ all of which occurred significantly more than expected (standardized residuals = 41.67, 33.68, and 11.09, respectively). In contrast, ‘AR poisoning’, ‘persecution’, ‘neurological distress (cause unknown)’, ‘contaminant exposure’, ‘attacked or caught by domestic pet’, ‘electrocution’, ‘collision with human infrastructure’, ‘disease’, ‘eye injury (cause unknown)’, and ‘collision with vehicle’ occurred less than expected (standardized residuals = -10.76, -10.66, -10.29, -10.11, -9.18, -8.53, -8.06, -7.60, -5.92, and -3.97, respectively), although we caution interpretation of the results for ‘AR poisoning’ (n = 0), ‘persecution’ (n = 1), and ‘neurological distress (cause unknown)’ (n = 5) due to small sample sizes.

**Table 2:** Number of owls admitted to rehabilitation between 2010 and 2021. Owls were admitted to two facilities that serve Texas, US (Last Chance Forever the Bird of Prey Conservancy and Wildlife Rescue & Rehabilitation, Inc.). The number in parenthesis represents the percentage of all owls admitted (n = 1620).

Species															
Admission Cause	Barn Owl		Barred Owl		Burrowing Owl		Great-horned Owl		Long-eared Owl		Screech Owl		Short-eared Owl		Total
	Adult	Juvenile	Adult	Juvenile	Adult	Juvenile	Adult	Juvenile	Adult	Juvenile	Adult	Juvenile	Adult	Juvenile	
Bodily Injury	22 (1.4)	8 (0.5)	127 (7.8)	10 (0.6)	7 (0.4)	0	234 (14.4)	12 (0.7)	3 (0.2)	0	49 (3.0)	5 (0.3)	1 (0.1)	0	478 (29.5)
Eye Injury	0	1 (0.1)	12 (0.7)	2 (0.1)	0	0	25 (1.5)	1 (0.1)	0	0	8 (0.5)	3 (0.2)	0	0	52 (3.2)
Collision with Vehicle	4 (0.2)	0	24 (1.5)	0	0	0	26 (1.6)	0	0	0	19 (1.2)	0	0	0	73 (4.5)
Collision with Human Infrastructure	1 (0.1)	0	7 (0.4)	0	2 (0.1)	0	14 (0.9)	0	0	0	5 (0.3)	0	0	0	29 (1.8)
Entrapment	5 (0.3)	0	24 (1.5)	1 (0.1)	1 (0.1)	0	65 (4.0)	3 (0.2)	0	0	1 (0.1)	1 (0.1)	0	0	101 (6.2)
Electrocution	0	0	4 (0.2)	0	0	0	18 (1.1)	0	0	0	2 (0.1)	0	0	0	24 (1.5)
Contaminant Exposure	0	0	0	0	0	0	6 (0.4)	0	0	0	0	1 (0.1)	0	0	7 (0.4)
Anticoagulant Rodenticides	0	0	0	0	0	0	0	0	0	0	0	0	0	0	0
Neurological Distress	0	0	0	0	1 (0.1)	0	2 (0.1)	0	0	0	1 (0.1)	1 (0.1)	0	0	5 (0.3)
Persecution	0	0	0	1 (0.1)	0	0	0	0	0	0	0	0	0	0	1 (0.1)
Attacked or Caught by Domestic Pet	0	0	1 (0.1)	1 (0.1)	0	0	1 (0.1)	0	0	0	11 (0.7)	3 (0.2)	0	0	17 (1.0)
Disease	2 (0.1)	18 (1.1)	8 (0.5)	0	0	0	3 (0.2)	6 (0.4)	0	0	3 (0.2)	1 (0.1)	0	0	34 (2.1)
No Apparent Injury	0	90 (5.6)	2 (0.1)	42 (2.6)	1 (0.1)	1 (0.1)	3 (0.2)	211 (13.0)	0	0	4 (0.2)	214 (13.2)	0	0	564 (34.8)
Undiagnosed	15 (0.9)	3 (0.2)	48 (3.0)	0	4 (0.2)	0	114 (7.0)	4 (0.2)	0	0	33 (2.0)	14 (0.9)	0	0	235 (14.5)
**Total**	49 (3.0)	113 (7.0)	257 (15.9)	57 (3.5)	16 (1.0)	1 (0.1)	511 (31.5)	237 (14.6)	3 (0.2)	0	132 (8.1)	243 (15.0)	1 (0.1)	0	1620

**Fig 3:**
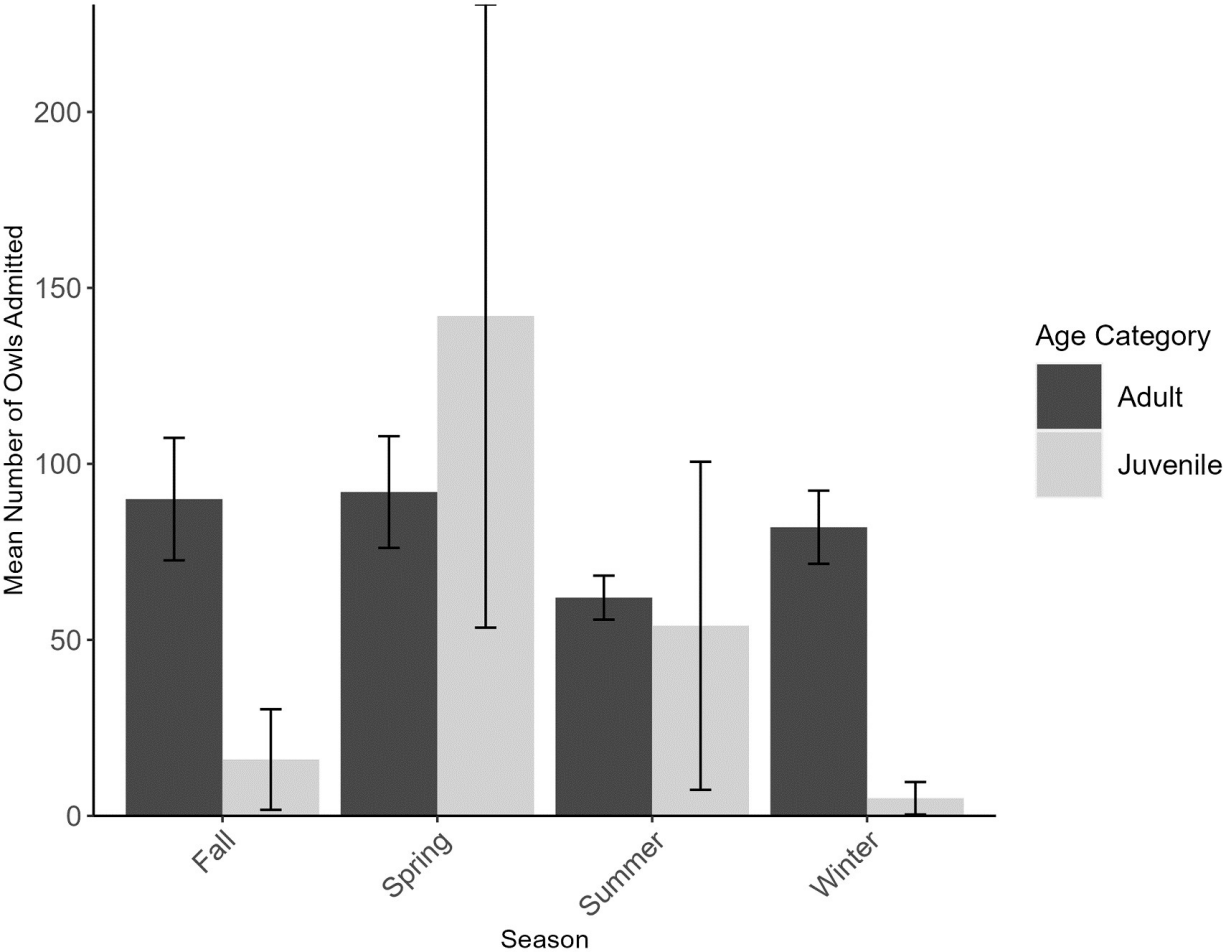
Number of owls admitted per species into rehabilitation between 2010 and 2021 (excluding healthy orphaned nestlings and fledglings admitted with ‘no apparent injury’). Owls were admitted to two facilities that serve Texas, US (Last Chance Forever the Bird of Prey Conservancy and Wildlife Rescue & Rehabilitation, Inc.). Dark grey bars represent adults and light grey bars represent juveniles.

When apparently healthy orphaned nestlings and fledglings categorized as ‘no apparent injury’ were omitted from the analysis, the number of admissions records decreased to 1,062 with an average of 88.5 individuals admitted per year (range: 34–149, SD: 42.54). Similarly to the results from the full dataset (i.e., the dataset that considered all admitted individuals), Great-horned Owls were the most frequently admitted species (n = 537, 50.56%), followed by Barred Owls (n = 272, 25.61%) and Screech Owls (n = 161, 15.16%) (Fig 3). The observed admittance counts per species were significantly different from expected (*X*^2^ = 289.63, df = 4, P<0.0001) with Barn, Great-horned, and Screech Owls being admitted more than expected (standardized residuals = 3.51, 3.28, and 11.57, respectively). In contrast, Barred and Burrowing Owls were admitted significantly less than expected (standardized residuals = -2.78 and -11.18, respectively).

**Fig 4:**
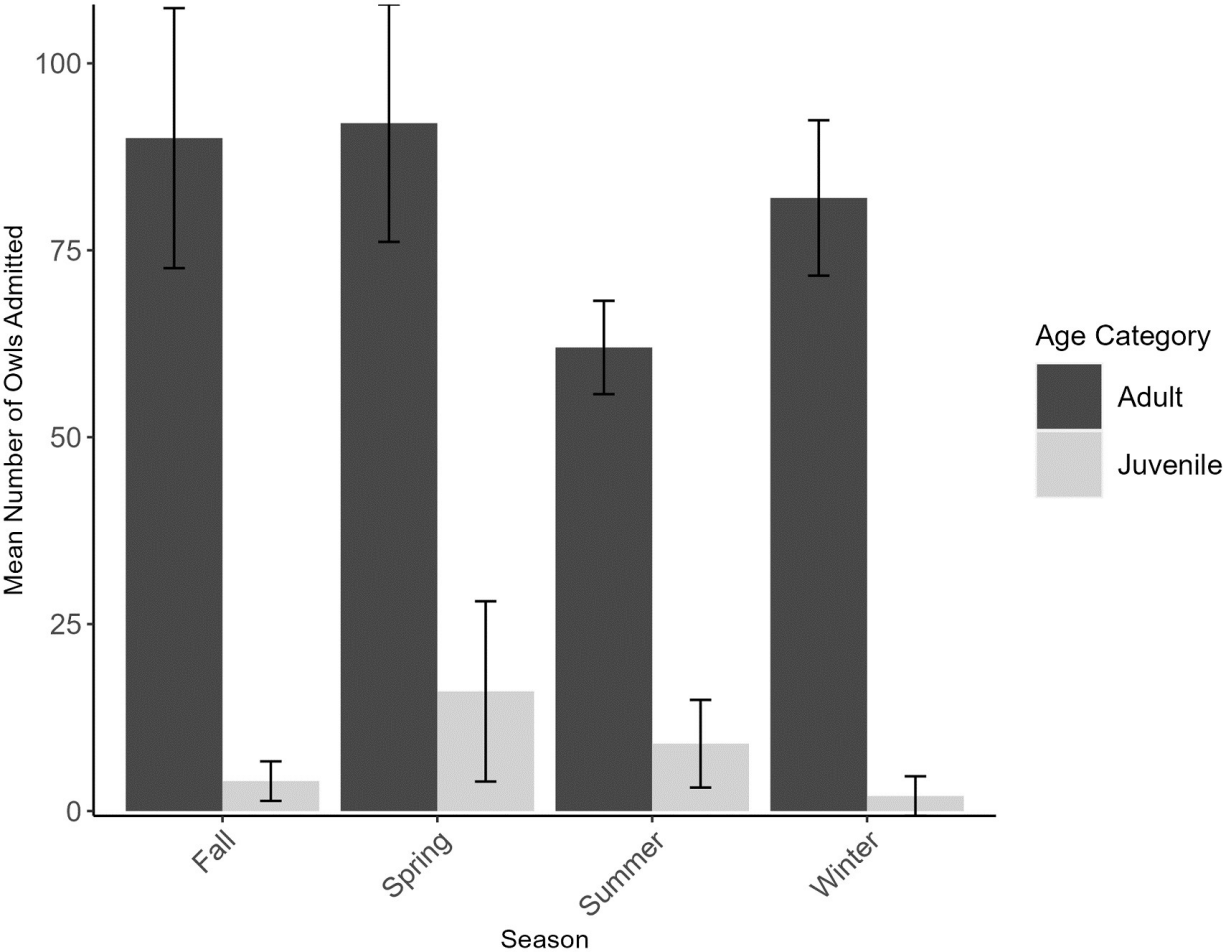
Number (mean ± 1SD) of owls admitted by season into rehabilitation between 2010 and 2021 (excluding healthy orphaned nestlings and fledglings admitted with ‘no apparent injury’). Owls were admitted to two facilities that serve Texas, US (Last Chance Forever the Bird of Prey Conservancy and Wildlife Rescue & Rehabilitation, Inc.) by season. Dark grey bars represent adults and light grey bars represent juveniles.

In accordance with the results from the full dataset, the majority of owls represented in the reduced dataset were admitted during spring (n = 325, 30.60%) (Fig 4). In addition, the percentage of admittances that occurred in the summer were similar between the two datasets (full dataset: n = 347, 21.42% *vs*. reduced dataset n = 212, 19.96%). However, when orphaned nestlings and fledglings categorized as ‘no apparent injury’ were omitted from the analysis, the percentage of admittances in fall and winter increased (fall full dataset: n = 310, 19.14% *vs*. fall reduced dataset: n = 273, 25.71%; winter full dataset: n = 260, 16.05% *vs*. winter reduced dataset: n = 252, 23.73%). Seasonal admittance counts calculated using the reduced dataset were significantly different from expected (*X*^2^ = 25.01, df = 3, P<0.0001) with more owls being admitted in spring (standardized residual = 3.65) and fall (standardized residual = 0.46) than expected, and fewer being admitted in summer (standardized residual = -3.28) and winter (standardized residual = -0.83).

Following the removal of healthy orphaned nestlings and fledglings admitted with ‘no apparent injury’ from the dataset, the most common cause of admittance was ‘bodily injury (cause unknown)’ (n = 478, 45.01%) and ‘undiagnosed’ (n = 235, 22.13%). These results are consistent with those obtained from the full dataset. Other relatively common causes of admittance included ‘entrapment with human infrastructure’ (n = 101, 9.51%) and ‘collision with vehicle’ (n = 73, 6.87%). In contrast, few admissions were due to ‘no apparent injury’ (n = 6, 0.56%), ‘contaminant exposure’ (n = 7, 0.70%), ‘neurological distress (cause unknown)’ (n = 5, 0.47%), and no owls were admitted due to ‘AR poisoning’. Counts for the different causes of admittance were significantly different from expected (*X*^2^ = 2,957.6, df = 13, P<0.0001). The greatest differences between observed and expected counts occurred for ‘bodily injury (cause unknown)’ and ‘undiagnosed’ (standardized residuals = 46.17 and 18.27, respectively). In contrast, ‘persecution’, ‘neurological distress (cause unknown)’, ‘no apparent injury’, and ‘contaminant exposure’ occurred less than expected (standardized residuals = -8.59, -8.14, -8.02, and -7.91, respectively), although we caution interpretation of these results due to small sample sizes.

### Anticoagulant rodenticide liver analysis

A total of 53 owl liver samples were screened for four FGARs and four SGARs (Great-horned Owl: n = 31, 58.49%; Barred Owl: n = 9, 16.98%; Barn Owl: n = 7, 13.21%; Screech Owl: n = 6, 11.32%). Of the 53 samples, nine were tested for an additional FGAR, coumachlor. Of all owls sampled, 27 (50.94%) were positive for at least one AR (67.74% of Great-horned Owls [n = 21], 33.33% of Barred Owls [n = 3], 28.57% of Barn Owls [n = 2], and 16.67% of Screech Owls [n = 1]). An SGAR, brodifacoum was the most frequently detected AR and occurred in 19 of the 53 owls sampled (35.85%), followed by bromadiolone (n = 17, 32.08%), difethialone (n = 13, 24.53%), and diphacinone (n = 3, 5.66%). Two of the nine liver samples (22.22%) assessed for coumachlor tested positive. In comparison, warfarin, chlorophacinone, coumatetralyl, and difenacoum were not detected in any samples. Eighteen of the 27 positive owls contained multiple ARs (66.67%). Of the 27 positive samples (n = 7) 25.93% were within the lethal range (i.e., toxic threshold) as defined by the standard reference value set forth by [41] at >100–200 ng/g ww. AR concentrations within the toxic threshold detected in owls ranged between 111.10–192.00 ng/g ww (Table 3).

**Table 3:** Results from anticoagulant rodenticide (AR) liver analysis for owls sampled in Texas, US between 2020 and 2022. ID Number annotates the source of the specimen and unique identifier as follows: Biodiversity Research and Teaching Collections, Texas A&M University (TCWC), Last Chance Forever the Bird of Prey Conservancy (LCF), The University of Texas at San Antonio (UTSA) and Wildlife Rescue & Rehabilitation Inc. (WRR). Unit of measurement is ng/g wet weight; limit of detection for each compound was 0.10 ng/g and limit of quantification for each compound was 1.00 ng/g. Negative results are indicated by a dash (―); n/a represents a compound not tested for.

Species/ID Number	Cause of Admission	Second Generation Anticoagulant Rodenticides (SGARs)			First Generation Anticoagulant Rodenticides (FGARs)	
		Brodifacoum	Bromadiolone	Difethialone	Diphacinone	Coumachlor
Barn Owl LCF 7	No Apparent Injury	―	―	―	―	n/a
Barn Owl LCF 13	Bodily Injury (wing)	―	76.70	―	―	n/a
Barn Owl LCF 16	Entrapment (barbed wire)	―	―	―	―	n/a
Barn Owl LCF 23	Bodily Injury (wing)	―	―	―	―	n/a
Barn Owl TCWC 29722	Undiagnosed	―	―	―	―	n/a
Barn Owl TCWC 30191	Undiagnosed	―	―	14.00	―	0.72
Barn Owl TCWC 30052	Undiagnosed	―	―	―	―	―
Barred Owl LCF 783 Yellow	Entrapment (barbed wire)	―	―	―	―	n/a
Barred Owl LCF 34 Pink	Vehicle Collision	―	―	―	―	n/a
Barred Owl LCF 33 Orange	Undiagnosed	―	*125.80	―	―	n/a
Barred Owl WRR 21–7775	Bodily Injury (wing)	―	―	―	―	n/a
Barred Owl WRR 21–7808	Bodily Injury (wing)	―	―	―	―	n/a
Barred Owl TCWC 29721	Undiagnosed	65.00	―	―	―	n/a
Barred Owl TCWC 29717	Undiagnosed	51.00	―	―	―	n/a
Barred Owl TCWC 30192	Undiagnosed	―	―	―	―	―
Barred Owl TCWC 30200	Undiagnosed	―	―	―	―	―
Great-horned Owl LCF 72 Orange	Undiagnosed	18.39	―	<1.00	―	n/a
Great-horned Owl LCF 6 Green	Bodily Injury (wing)	15.43	7.02	<1.00	―	n/a
Great-horned Owl LCF 201 Orange	Bodily Injury (wing)	―	―	―	―	n/a
Great-horned Owl LCF 69 Orange	Entrapment (barbed wire)	―	―	―	―	n/a
Great-horned Owl LCF 47 Orange	Bodily Injury (wing)	―	―	―	―	n/a
Great-horned Owl LCF 99 Orange	Entrapment (barbed wire)	―	―	―	―	n/a
Great-horned Owl LCF 61 Orange	Undiagnosed	―	―	―	―	n/a
Great-horned Owl LCF 1 Blue	Undiagnosed	6.84	*117.99	<1.00	1.95	n/a
Great-horned Owl LCF 27 Pink	Bodily Injury (wing & leg)	2.99	<1.00	―	―	n/a
Great-horned Owl LCF 29 Pink	Bodily Injury (wing)	―	5.51	―	2.52	n/a
Great-horned Owl LCF 33 Pink	Bodily Injury (wing)	1.99	32.58	―	―	n/a
Great-horned Owl LCF 16 Orange	Undiagnosed	―	―	―	―	n/a
Great-horned Owl LCF 1 Pink	Undiagnosed	8.28	*136.77	―	*116.43	n/a
Great-horned Owl LCF 38 Orange	Bodily Injury (wing)	―	―	<1.00	―	n/a
Great-horned Owl LCF 58 Orange	Bodily Injury (wing)	21.81	16.08	<1.00	―	n/a
Great-horned Owl LCF 27 Orange	Electrocution	20.81	―	―	―	n/a
Great-horned Owl LCF 7 Orange	Undiagnosed	―	―	―	―	n/a
Great-horned Owl WRR 21–7022	Bodily Injury (wing)	*111.10	*117.60	5.30	―	n/a
Great-horned Owl WRR 21–8493	Electrocution	*178.70	―	*196.00	―	n/a
Great-horned Owl WRR 21–8014	Entrapment (fence)	―	―	―	―	n/a
Great-horned Owl LCF 71	Electrocution	―	―	―	―	n/a
Great-horned Owl WRR 73	Bodily Injury (wing)	65.80	―	44.10	―	n/a
Great-horned Owl WRR 74	Electrocution	65.80	25.50	3.80	―	n/a
Great-horned Owl WRR 75	Bodily Injury (wing)	8.40	41.60	―	―	n/a
Great-horned Owl WRR 76	Bodily Injury (wing)	3.40	48.80	30.10	―	n/a
Great-horned Owl TCWC 29294	Undiagnosed	24.70	―	1.10	―	n/a
Great-horned Owl TCWC 20295	Undiagnosed	66.50	55.70	26.50	―	n/a
Great-horned Owl TCWC 30158	Undiagnosed	―	*144.00	―	―	―
Great-horned Owl TCWC 30160	Undiagnosed	―	―	―	―	―
Great-horned Owl TCWC 30161	Undiagnosed	―	*192.00	―	―	0.59
Great-horned Owl TCWC 30159	Undiagnosed	―	50.00	―	―	―
Screech Owl LCF 20	Bodily Injury (wing)	―	―	―	―	n/a
Screech Owl LCF 31	Attacked by Cat	―	―	―	―	n/a
Screech Owl UTSA 41	Undiagnosed	―	―	―	―	n/a
Screech Owl WRR 72	Bodily Injury (wing)	4.50	―	―	―	n/a
Screech Owl TCWC 29780	Undiagnosed	―	―	―	―	n/a
Screech Owl TCWC 29779	Undiagnosed	―	―	―	―	―

*Denotes lethal levels of >100 ng/g ww [41].

## Discussion

This study provides important new insights into the anthropogenic threats faced by owls in Texas, a region with a high species richness of owls which lacked assessments of anthropogenic threats owls face, hindering the development of informed conservation plans. In support of our hypotheses, Great-horned Owls were the most often admitted species and admittance rates for all species combined were highest in the spring due to an influx of orphaned juveniles. By using data from admittance records from two rehabilitation centers that receive owls from across Texas coupled with AR liver screening we demonstrated, in partial support of our hypothesis, that the leading causes of admittance when all owls were considered were ‘no apparent injury’, ‘bodily injury (unknown cause)’ and ‘undiagnosed’, and that this pattern was consistent amongst species. When apparently healthy orphaned nestlings and fledglings categorized as ‘no apparent injury’ were omitted from the analysis, the leading cause of admittances were ‘bodily injury (unknown cause)’ and ‘undiagnosed’. Of the 53 owls screened for ARs, 50.94% tested positive, suggesting that AR exposure is prevalent in Texas.

Great-horned Owls were the most admitted species, followed by Screech Owls and Barred Owls. These three species represent the most common owls in the region [34], likely explaining our results. Similarly, numerous other studies have shown Great-horned Owls, Screech Owls, and Barred Owls to be the most commonly admitted species to rehabilitation centers in North America [6, 17, 31]. In addition to being relatively common in Texas, all three species are found in urban areas where detection rates are likely higher, which may in part explain their high admittance rates. Certainly, our results suggest that Great-horned and Screech Owls were admitted more often than expected by their respective population sizes. At the same time, owls in urban areas may experience increased risk from human-caused threats associated with the built landscape (e.g., bird-building collisions, electrocutions, exposure to contaminants [42]) resulting in admittance to rehabilitation centers [24]. Despite these ideas, further study is needed to elucidate the effects of urbanization on admittance rates of owls both within and beyond Texas.

Based on population estimates, Burrowing Owls were admitted less than expected. In Texas, Burrowing Owls are concentrated in the western part of the state where they reside on private lands in rural areas [43]. In addition, the population is partially migratory with a decreased population size during the breeding season. As a result, detection of injured Burrowing Owls may be less than for other species represented in this study which occur across a larger area within the state and are non-migratory. It is also possible that Burrowing Owls did not face to the same extent anthropogenic threats underpinning admittance of other species represented in this study. Given Burrowing Owls are considered a species of conservation concern in Texas, additional study exploring threats they face in Texas is warranted.

The leading cause of admittance for all owls combined was ‘no apparent injury’, accounting for 34.81% of admittances. Five hundred and fifty-eight individuals (558/564) admitted under this category were juveniles admitted as uninjured orphans or displaced individuals. Similarly, orphaned individuals accounted for 17% of owls admitted to rehabilitation centers in the United Kingdom [24] and 53% of Little Owls (*Athene noctua*) admitted to a rehabilitation center in Spain [26]. Collectively, these results suggest that young birds with no apparent injuries are often admitted to wildlife rehabilitation centers, likely when they are found climbing on branches post-fledging, mistaken as being abandoned, or when they fall from their nest. The high admittance rates in spring resulting from an influx of juveniles corroborate this idea and are further supported by results from Wendell et al. [30] who demonstrated high admittance rates of juvenile owls during spring and summer.

When apparently healthy orphaned nestlings and fledglings categorized as ‘no apparent injury’ were omitted from the analysis the leading cause of admittance accounting for 45.01% of the remaining owls was ‘bodily injury’, assigned to individuals with broken bones and miscellaneous injuries where the cause was unknown. Similarly, Wendell et al. [30] described general trauma to be the number one cause of admission and cause of mortality for owls admitted to a facility in Colorado, US. An additional 22.13% of owls in our reduced dataset were admitted under the ‘undiagnosed’ category due to birds showing signs of e.g., unexplained lethargy, dehydration, malnutrition, or emaciation. Collectively, these results showcase the challenges of providing a definitive diagnosis or main cause of admittance for owls entered into rehabilitation. This may be especially prevalent when owls have multiple injuries and complications resulting in a general category (e.g., bodily injury [causes unknown]) being assigned to encompass all apparent injuries. In addition, animals brought to wildlife rehabilitation centers often are not accompanied with relevant information about the potential cause of admittance, prohibiting accurate diagnosis [6].

Under scenarios where clear diagnoses could be made, the leading causes of admittances were ‘entrapment with human infrastructure’ or ‘collision with vehicles’ accounting for 6.23% and 4.51% of all admitted owls, respectively. Similarly, two of the leading causes of admittance of owls into rehabilitation with known causes of injury in Alabama, US were collisions with vehicles and entanglement with barbed wire [6]. Roadkill studies in North America provide further evidence that owls are highly susceptible to vehicle collisions [11] and suggest that owl-vehicle collisions may occur when owls are attracted to roads due to the availability of small mammals [44] or because of the presence of suitable perches (e.g., fence posts). The potential for owls to be attracted to fence posts may also underlie their susceptibility to entrapment in barbed wire. While ‘electrocution’ and ‘collision with human infrastructure’ accounted for relatively few individuals in this study, the incidences add to existing evidence that owls are susceptible to electrocution via overhead electrical systems [8, 45] and building/window collisions [24, 45].

While the rehabilitation data did not reveal any incidences of AR exposure, liver analysis suggested high AR exposure rates of owls in Texas; of the 53 owls screened for ARs, 50.94% tested positive for at least one compound while 18 (33.96%) individuals were exposed to multiple ARs. These results showcase that, in the absence of diagnostic testing, rehabilitation admittance data may underestimate rates of AR exposure. Our results of exposure to at least one AR are similar to published exposure rates for owls found dead or admitted into rehabilitation centers in North America (Barn Owls: 33–62% [16, 46]; Great-horned Owls: 65–100% [17, 18, 22, 47]; Barred Owls: 75–96% [17, 18, 22] Eastern Screech Owls: 87% [22]) and suggest ARs are commonly used to control rodent pests consumed by owls. In similarity to previous studies in North America [18, 46], the most commonly detected ARs were SGARs brodifacoum and bromadiolone found in 35.85% and 32.08% of owls tested, respectively. A third SGAR, difethialone was detected in 24.53% of owls tested. Sales of bait products containing brodifacoum, bromadiolone and difethialone were discontinued for general and residential consumers in 2011 by the Environmental Protection Agency (EPA [48]), although their use by pest control operators and the agricultural sector is still permitted. The prevalence of these compounds in liver samples in this study suggests continued and widespread use resulting in lethal levels of exposure in seven (13.21%) of the owls sampled in this study. This raises concerns about other at-risk species including numerous species of owls of conservation concern in Texas that consume rodent pests (e.g., Burrowing Owls, Short-eared Owls, Long-eared Owls, and Mexican Spotted Owls [*Strix occidentalis lucida*] [34]).

By coupling rehabilitation data with clinical assessments of ARs, we have expanded our understanding of threats owls face in Texas. However, we acknowledge the limitations associated with our approach. First, owls sampled from wildlife rehabilitation centers represent a nonrandom sample of the population, and thus the AR exposure rates presented are unlikely representative of population level exposure rates. Second, admittance may be biased where individual size and injury type affects the probability of detection. For example, larger species like Great-horned Owls may be more readily detected, while grounded juvenile owls in urban areas or owls hit by vehicles in areas of high traffic may be more likely to be reported than individuals persecuted away from public view. Third, our study did not consider exposure to other contaminants known to negatively affect raptors (e.g., heavy metals, pesticides, polychlorinated biphenyls [49]). Nevertheless, our study provides important insight into the anthropogenic threats owls face, when they are at risk, and what species are at risk in an important region for these birds. In so doing, our results can be used to inform conservation plans that aim to minimize further losses of owls and the associated ecosystem services they provide [2–4]. This is especially relevant in Texas where rapid urbanization [32] is expected to increase anthropogenic threats to owls (e.g., vehicle and building collisions, entanglement with materials, electrocution, contaminant exposure, and other related trauma to owls [24]). Future efforts should consider systematic non-lethal sampling of owls for ARs and a broader suite of contaminants. Continued monitoring of ARs in free living populations would further aid mitigation strategies and conservation management plans.

## Supporting information

S1 Table Population estimates for owl species in Texas, US derived from the partners in flight database. (PDF)
