# Supplementary material for: Anthropogenic threats to owls: Insights from rehabilitation admittance data and rodenticide screening in Texas
Source: PLoS One. 2023 Aug 4;18(8):e0289228. doi: 10.1371/journal.pone.0289228 (PMC10403058; doi:10.1371/journal.pone.0289228)
Supplement: S1 Table — (PDF) [file pone.0289228.s001.pdf]

S1 Table. Population estimates for owl species in Texas, US derived from the Partners in Flight Database

Species Common Name	Population Estimate
Barn Owl	43,000
Barred Owl	290,000
Burrowing Owl	140,000
Great-horned Owl	420,000
Eastern Screech Owl	60,000

Data were retrieved from: Bird Conservancy of the Rockies, Avian Conservation Assessment and Population Estimates Databases; 2023 [cited 2023 July 6]. Partners in Flight Databases: Population Estimates [Internet]. Available from: https://pif.birdconservancy.org/population-estimate-database-scores/
